# Establishing zebrafish as a model to study the anxiolytic effects of scopolamine

**DOI:** 10.1038/s41598-017-15374-w

**Published:** 2017-11-08

**Authors:** Trevor J. Hamilton, Adam Morrill, Kayla Lucas, Joshua Gallup, Megan Harris, Meghan Healey, Taylor Pitman, Melike Schalomon, Shannon Digweed, Martin Tresguerres

**Affiliations:** 1 Department of Psychology, MacEwan University, Edmonton, AB T5J 4S2 Canada; 2 Neuroscience and Mental Health Institute, University of Alberta, Edmonton, AB T6G 2H7 Canada; 3 Scripps Institution of Oceanography, University of California San Diego, La Jolla, CA 92093 USA

## Abstract

Scopolamine (hyoscine) is a muscarinic acetylcholine receptor antagonist that has traditionally been used to treat motion sickness in humans. However, studies investigating depressed and bipolar populations have found that scopolamine is also effective at reducing depression and anxiety symptoms. The potential anxiety-reducing (anxiolytic) effects of scopolamine could have great clinical implications for humans; however, rats and mice administered scopolamine showed increased anxiety in standard behavioural tests. This is in direct contrast to findings in humans, and complicates studies to elucidate the specific mechanisms of scopolamine action. The aim of this study was to assess the suitability of zebrafish as a model system to test anxiety-like compounds using scopolamine. Similar to humans, scopolamine acted as an anxiolytic in individual behavioural tests (novel approach test and novel tank diving test). The anxiolytic effect of scopolamine was dose dependent and biphasic, reaching maximum effect at 800 µM. Scopolamine (800 µM) also had an anxiolytic effect in a group behavioural test, as it significantly decreased their tendency to shoal. These results establish zebrafish as a model organism for studying the anxiolytic effects of scopolamine, its mechanisms of action and side effects.

## Introduction

Scopolamine (hyoscine) is a high-affinity muscarinic acetylcholine receptor antagonist that has traditionally been used to treat motion sickness and nausea in humans^1^ and is also commonly used in memory research in laboratory animals^2,3^. In addition to the general feelings of nausea and discomfort that accompany motion sickness, prolonged affliction can result in symptoms such as apathy and depression^1^. Because scopolamine may also be effective in treating these additional symptoms, it is a potential antidepressant and anxiety-reducing (anxiolytic) drug. In clinically depressed and bipolar populations scopolamine was effective at reducing depressive symptoms and acting as an anxiolytic^4,5^ and has also been reported to reduce anxiety during cocaine withdrawal^6^. However, before it can be prescribed as an anxiolytic drug in humans, it is necessary to characterize its mechanism of action and potential side effects. These tasks are complicated because scopolamine research using rodent models has yielded conflicting results. In direct contrast to humans, rats administered scopolamine display increased anxiety (anxiogenic responses) in behavioural tests such as the open-field and light/dark test^7,8^. Although the exact reasons are unknown, the anxiogenic effects of scopolamine in rodents may be due to off-target effects such as the disruption of hippocampal cholinergic function, leading to a loss of the ability to process the amount of potential threat a situation poses^9^ and alteration of contextual processing^10,11^.

The zebrafish (*Danio rerio*) is becoming an increasingly popular model for research on affective disorders and pharmacology due to their salient behavioural phenotypes, compatibility with rodent experimental paradigms, and general efficiency for use as a research organism^12^. Because there is a wide range of behavioural tests available for zebrafish, they are commonly used to study the underlying neural mechanisms of anxiety and pharmacological compounds that alter these behaviours with many of the findings being translatable to mammals and humans^12^. In zebrafish, scopolamine has been characterized as an amnestic (memory reducing) agent, and is commonly used in combination with nootropic and memory-enhancing drugs to study memory formation and rescue. For example, scopolamine administered prior to a one-trial inhibitory avoidance paradigm resulted in decreased response to the learned stimulus, which was interpreted as scopolamine altering memory formation for the aversive stimulus^13^. In another study, zebrafish treated with scopolamine demonstrated impaired acquisition and retention of the avoidance response in a passive-avoidance paradigm^14^. However, behavioural tests designed to study memory could be affected by a potential anxiolytic effect of scopolamine. Thus, the lack of memory acquisition^14^ and memory retention^13^ could be due to decreased anxiety during the training phase, resulting in lack of fear to the noxious stimulus during testing. To date, only one study tested the potential anxiolytic effect of scopolamine on zebrafish^15^. Based on a lack of effect on light-avoidance behaviour, that study concluded scopolamine had no direct affect on zebrafish anxiety but did reduce the anxiolytic action of physostigmine, an acetylcholinesterase inhibitor. However, scopolamine also has a mydriatic effect on the pupil, causing it to remain dilated in bright conditions^16^. This issue could confound results in the light-avoidance test as well as in the light/dark test, which is one of the most common methods to test anxiety in fish^17–19^.

The novel approach test is another method to investigate the effects of drugs on zebrafish behaviour, but it does not rely on the ability to detect light or dark illumination. In the novel approach test, a zebrafish is placed in a circular arena with a novel object in the center and time spent investigating the object is quantified. In this test, zebrafish treated with well established anxiolytic drugs such as ethanol spend significantly more time close to the novel object rather than near the arena wall^12,20^. Yet another behavioural tests that is not light-dependent is the “novel tank diving response”^21^, whereby decreased anxiety-like behaviour is proportional to the time spent swimming in the top of the water column. Finally, zebrafish anxiety-like behaviour can also be studied using group behaviour paradigms. In one of the simplest and most robust tests, the tightness of a shoal of fish is proportional to zebrafish anxiety, and is decreased by anxiolytics^22,23^. To eliminate any potential confounds in perception of brightness and resultant light-avoidance caused by scopolamine, we used the novel approach test, novel tank diving test, and shoaling rather than a light/dark test to examine the potential anxiolytic effect of scopolamine in zebrafish.

## Results

### Effects of Ethanol in the Novel Approach Test

**Figure 1:**
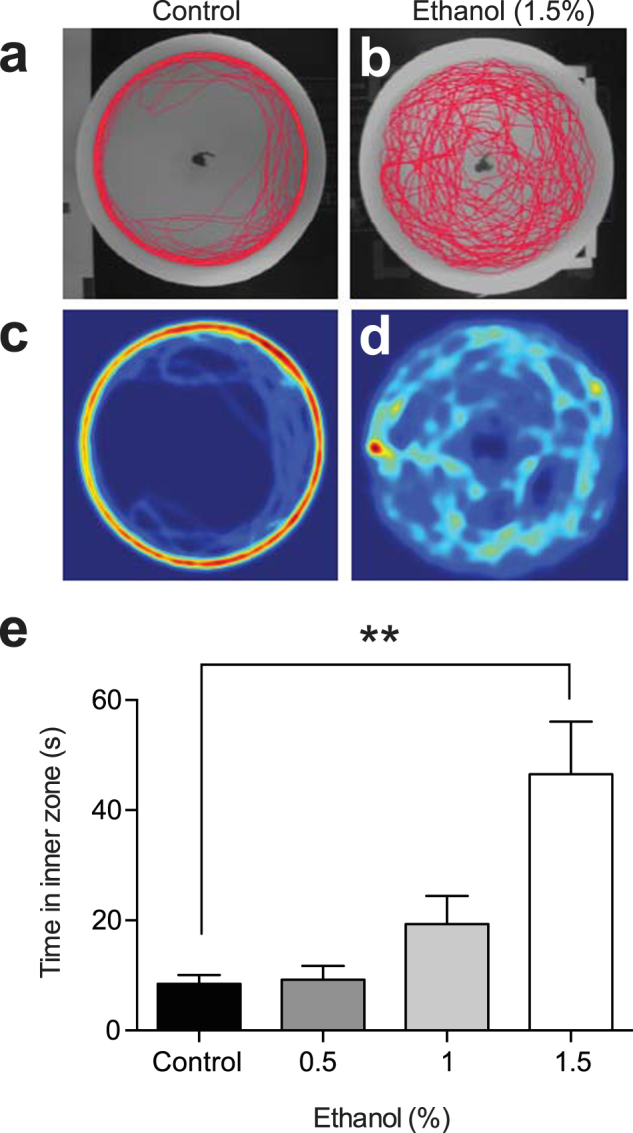
Ethanol decreases anxiety in the novel approach test. (**a**) Trackplot of an individual control zebrafish behaviour in the 5 minute trial. (**b**) Trackplot of an individual zebrafish exposed to 1.5% ethanol. (**c**) Heatmap (coloured representation of zebrafish location over the trial) for the same fish shown in (**a**). (**d**) Heatmap for the same fish shown in (**b**). (**e**) Time fish spent in the inner zone containing the novel object for different doses of ethanol. **P < 0.01, Kruskal-Wallis test with Dunn’s multiple comparison post hoc test.

In order to validate the novel approach test for anxiety-like behaviour studies, we first tested the effects of 0.5, 1.0, and 1.5% ethanol on zebrafish behaviour. Ethanol had a dose-dependent effect on time spent in the inner zone near the object (H(4) = 15.27, P = 0.0016, Kruskal-Wallis test), reaching statistically significant differences at 1.5% ethanol (46.5 ± 9.6 s, n = 18) compared to controls (8.5 ± 1.6 s, n = 18)(P < 0.01, Dunn’s multiple comparison test) (Fig. 1). Fish treated with 1.5% ethanol also demonstrated reduced locomotor activity, measured as distance traveled and immobility (Table 1). The increased time spent near the novel object reflects an anxiolytic effect of ethanol in our test, as zebrafish are typically neophobic and engage in thigmotaxic behaviour when anxious^24^. These results validate the novel approach test as an appropriate method of measuring anxiety-like behaviour in zebrafish.

**Table 1:** Distance traveled and immobility for control and ethanol-dosed zebrafish in the novel approach test.

	Distance Travelled (cm)	Immobility (s)
Control (n = 18)	3673 ± 234.7	16.06 ± 7.677
0.5% Ethanol (n = 16)	3231 ± 175.7	3.827 ± 3.048
1.0% Ethanol (n = 15)	2793 ± 561.3	22.70 ± 16.09
1.5% Ethanol (n = 18)	2647 ± 339.7	76.46 ± 22.63**

There was a significant difference between controls and 1.5% ethanol for immobility (**P < 0.01) (Kruskal-Wallis test with Dunn’s multiple comparison post hoc test).

### Effects of Scopolamine in the Novel Approach Test

**Figure 2:**
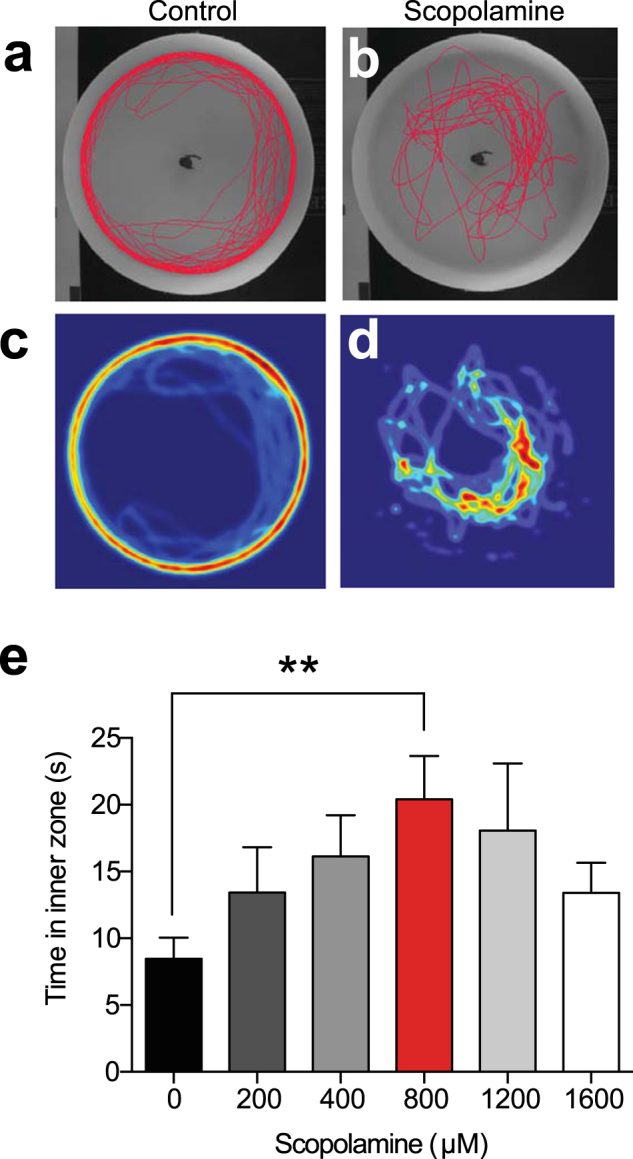
Scopolamine decreases anxiety in the novel approach test. (**a**) Trackplot of an individual control zebrafish behaviour in the 5 minute trial. (**b**) Trackplot of an individual zebrafish exposed to 800 μM scopolamine. (**c**) Heatmap (coloured representation of zebrafish location over the trial) for the same fish shown in (**a**). (**d**) Heatmap for the same fish shown in (**b**). (**e**) Time fish spent in the inner zone containing the novel object for different doses of scopolamine. **P < 0.01, Kruskal-Wallis test with Dunn’s multiple comparison post hoc test.

To determine whether scopolamine affects zebrafish anxiety, we administered scopolamine for 30 minutes, and then placed the fish in the novel approach test. Similar to ethanol, scopolamine had a dose-dependent effect on time spent in the inner zone near the object, with an EC_50_ of 251.5 μM, and the 800 µM dose having a maximum effect (20.4 ± 3.252 s, n = 19). The 800 µM dose was significantly different from controls (8.468 ± 1.594 s, n = 18) (H(6) = 11.55, P = 0.0415, Kruskal-Wallis test) (Fig. 2). Scopolamine had no significant effects on locomotion (distance travelled and immobility) (Table 2).

**Table 2:** Distance traveled and immobility for control and scopolamine-dosed zebrafish in the novel approach test.

	Distance travelled (cm)	Immobility (s)
Control (n = 18)	3673 ± 234.7	16.06 ± 7.677
200 µM scopolamine(n = 18)	4115 ± 228.4	6.742 ± 3.137
400 µM scopolamine (n = 16)	3738 ± 287.2	1.403 ± 0.8007
800 µM scopolamine (n = 19)	3926 ± 377.6	8.051 ± 3.78
1200 µM scopolamine (n = 16)	2855 ± 183.1	4.023 ± 2.275
1600 µM scopolamine (n = 18)	3821 ± 263.1	10.08 ± 6.647

There were no significant differences between controls and scopolamine for distance travelled and immobility for all scopolamine doses.

### Effects of Scopolamine in the Novel Tank Diving test

**Figure 3:**
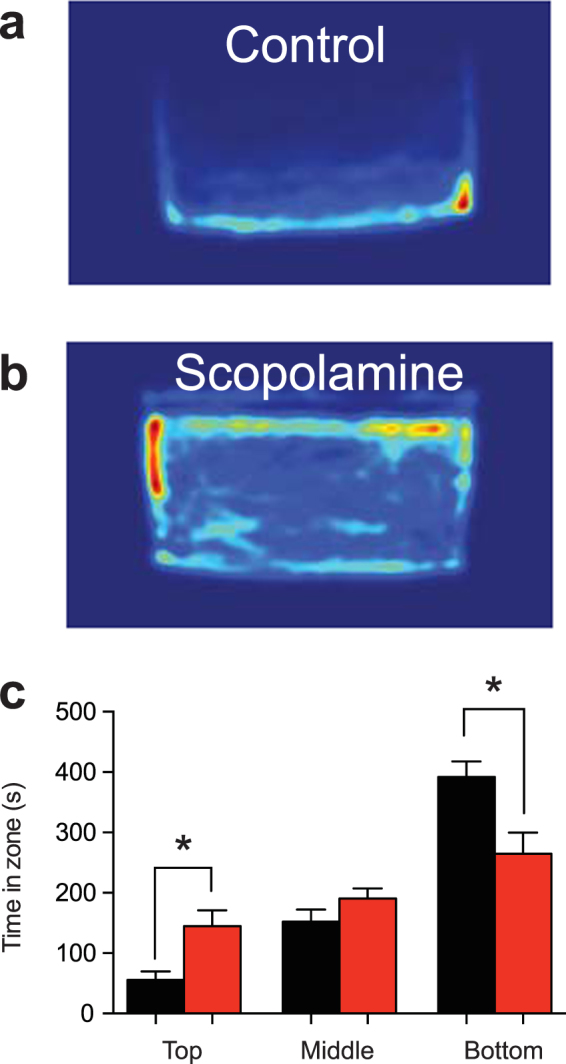
Scopolamine decreases anxiety in the novel tank diving test. (**a**) Heatmap of an individual control zebrafish behaviour in the 10 minute trial. (**b**) Heatmap of an individual zebrafish exposed to 800 μM scopolamine. (**c**) Time fish spent in each zone for an 800 μM dose of scopolamine (red bars) compared to controls (black bars). *P < 0.05, unpaired t-test.

The novel tank diving test is a robust measure of anxiety in zebrafish, with anxiolytic drugs causing zebrafish to spend more time in the upper zone of the tank^21,25^. Zebrafish were exposed to 800 μM scopolamine, which was found to be the most effective in the novel approach test, and subjected to the novel tank diving test. Scopolamine significantly decreased the time spent in the bottom zone (Fig. 3)(control: 391.8 ± 25.7 s, n = 10; scopolamine: 264.6 ± 35.2 s, n = 13; unpaired t-test, t = 2.756, df = 21, P = 0.0118) and significantly increased the time spent in the top zone (control: 55.8 ± 13.8 s, n = 10; scopolamine: 144.9 ± 26.3 s, n = 13; unpaired t-test, t = 2.745, df = 21, P = 0.0121). Additionally, scopolamine had no effect on the time spent in the middle zone (control: 152.4 ± 19.9 s, n = 10; scopolamine: 190.8 ± 16.5, n = 13; unpaired t-test, t = 1.498, df = 21, P = 0.1490). Altogether, these results indicate a strong anxiolytic effect of scopolamine at a dose of 800 µM.

### Effects of Scopolamine on Shoaling

**Figure 4:**
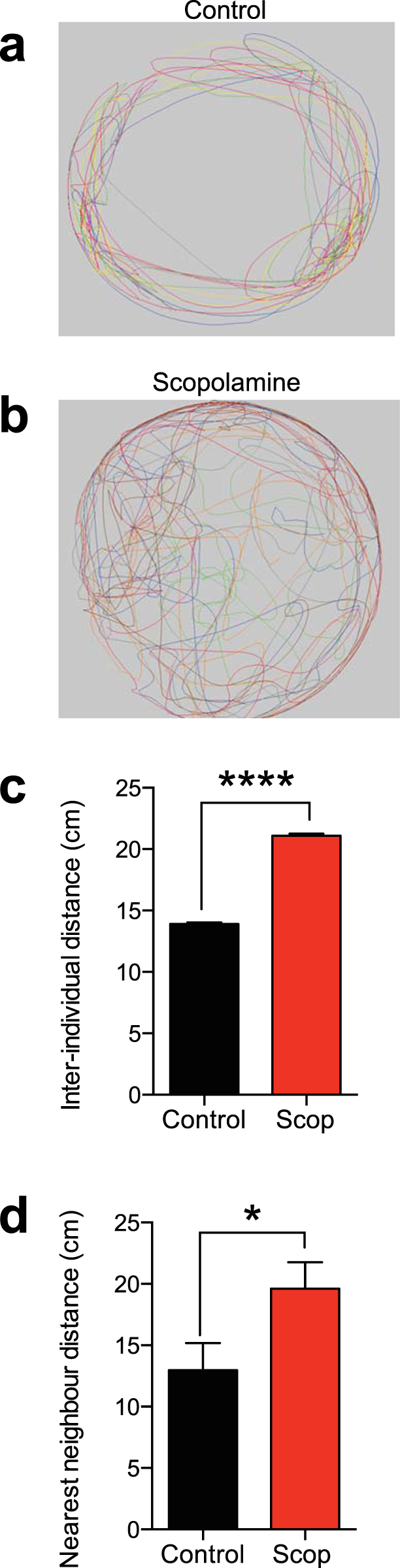
Scopolamine decreases anxiety in a test of group behaviour. (**a**) Trackplot of a group of 6 control zebrafish for the first 30 seconds of the trial. Each coloured line represents an individual fish. (**b**) Trackplot of a group of 6 zebrafish dosed with 800 μM scopolamine. (**c**) Scopolamine (800 μM) increased the inter-individual distance in groups of zebrafish. (**d**) Scopolamine (800 μM) increased the nearest neighbour distance in groups of zebrafish. *P < 0.05, ****P < 0.0001, unpaired t-test.

Lastly, we tested the effect of 800 µM of scopolamine on groups of 6 fish in a shoaling test, in which the tightness of the shoal is a measure of anxiety^22,23^. Again consistent with an anxiolytic effect, scopolamine-treated zebrafish demonstrated significantly greater inter-individual distance compared to controls (Fig. 4)(control: 13.9 ± 0.1 cm, n = 6; scopolamine: 21.0 ± 0.1 cm, n = 6, t = 35.17, df = 10, unpaired t-test, P < 0.0001). Scopolamine also significant increased the nearest neighbor distance (Fig. 4) (control: 12.96 ± 2.2 cm, n = 6; scopolamine: 19.6 ± 2.1 cm, n = 6; Mann-Whitney U-test, two-tailed, P = 0.0411).

## Discussion

This study demonstrates the anxiolytic effect of scopolamine on zebrafish in three tests of anxiety-like behaviour. First, we validated the novel approach test pharmacologically with ethanol. Consistent with recent research^20^ and with its established anxiolytic effects in mammals^26^, ethanol increased the time zebrafish spent in the inner zone near the object. Because scopolamine had the same effect as ethanol in the novel approach test, we conclude it also acts as an anxiolytic in zebrafish. This anxiolytic effect was seen most prominently at a dose of 800 μM. This finding was further validated by investigating shoaling and tank diving behaviour, where 800 μM scopolamine also caused a significant decrease in behaviours normally associated with anxiety in zebrafish. All three of the tests used in this study control for the potential light-avoidance confound in previous research, and each test yielded congruent results. Our findings suggest that scopolamine is an effective anti-anxiety agent in zebrafish, and that zebrafish are a valid model organism for studying the effects of therapeutic anxiolytic drugs.

There are many well-validated tests of anxiety-like behaviour in zebrafish (for review see references^12,27^) yet to date there has only been one study that examined the effects of scopolamine on anxiety in zebrafish. Cho and colleagues (2011)^15^ placed zebrafish in a 2-L tank with a light source suspended above the water and anxiety was measured as light-avoidance behavior. Fish were dosed with either 100 or 200 μM scopolamine or acetylcholinesterase inhibitor physostigmine, and it was found that only zebrafish treated with physostigmine showed the anxiolytic behavior (preferring to swim near the top of the tank), whereas fish administered either concentration of scopolamine did not differ from controls. When both drugs were administered at the same time, the anti-anxiety effects of physostigmine were seemingly suppressed by scopolamine, as these fish avoided the light source by spending more time at the bottom of the tank. In contrast to our study, the findings of Cho and colleagues (2011)^15^ suggest that scopolamine counters the anxiolytic effect of physostigmine and may itself cause light avoidance in zebrafish. However, it is important to consider the impact of potential drug side effects when using certain behavioral paradigms, as scopolamine is known to have a mydriatic effect on the pupil causing it to remain dilated in bright conditions^16^. Previous animal research showing anxiogenic effects of scopolamine in light-dark and light-avoidance tests could be problematic in that they assume the zebrafish^15^ or rats^7,8^ are photophobic and that avoidance of brighter areas is a sign of anxiety. While this may be a valid assumption in most other pharmacological studies, the specific mydriatic effect that scopolamine has on the pupil could cause non-anxious animals to avoid brighter areas purely as a result of physical discomfort especially when the animals are immersed in a drug solution rather than injected with the drug. The validity of previous animal research describing this drug as having anxiogenic effects could be negatively impacted by this potential confound, as it is important to select appropriate behavioural paradigms when measuring scopolamine’s effect on anxiety. To eliminate any potential confounds in perception of brightness and resultant light-avoidance, we used a novel approach test, a novel tank diving test, and a shoaling test instead of a light/dark test to examine the effect of scopolamine and found a consistent anxiolytic effect at a much higher dose of scopolamine (800 μM).

The potential anxiolytic properties of scopolamine should also be considered when using memory paradigms involving fear and anxiety. Scopolamine is undoubtedly an amnesic agent in zebrafish, confirmed by non-anxiety/fear based tasks such as the T-maze^2^ and virtual object recognition task^28^, however, future memory studies should carefully consider the dose, duration, and experimental test to avoid any crosstalk with anxiety-reducing effects of scopolamine. In the current study, exposure to 800 μM scopolamine for 30 minutes significantly affected novel stimuli processing, tank diving, and group behaviour. Thus, we advise future research using scopolamine as an amnesic agent to perform complementary anxiety tests to validate their memory tests. For example, memory formation was decreased with an inhibitory avoidance to electric shock test in zebrafish dosed with 200 μM scopolamine for one hour^13^. Importantly, these researchers also conducted an anxiety test with the same dose of scopolamine and found no effects in the novel tank diving test, suggesting anxiety was not affected and that the effects of scopolamine in the avoidance test were indeed due to a true memory deficit. On the other hand, zebrafish exposed to 400 μM scopolamine for one hour demonstrated memory impairment in a fear conditioning test^29^. However, as shown in the current study, this scopolamine concentration and exposure time can have anxiolytic effects, making it impossible to attribute the lack of memory to an amnesic or anxiolytic effect, or both.

## Conclusion

This study indicates that scopolamine induces anxiolytic effects in zebrafish. Specifically, scopolamine increased the time zebrafish spent near the novel object in the novel approach test, increased the time fish spent in the top zone of the novel tank diving test, and decreased shoal cohesion. All of these effects are consistent with well-established anxiolytic drugs in other zebrafish studies^12,20,27^, and cannot be attributed to unwanted side effects on the pupil that might affect light avoidance. Because the anxiolytic effect of scopolamine on zebrafish behaviour is consistent with what is seen in humans^4–6^, we conclude zebrafish is a suitable model organism to test potential anxiolytic compounds for eventual human use.

## Methods

### Animals and Housing

Adult wild-type (short fin) zebrafish were obtained from a commercial supplier (Big Al’s Aquarium and Fish Supply, Edmonton, AB), were minimally 6 months of age (n = 200), and gender was not determined. Fish were housed in an Aquatic Habitats (AHAB, Aquatic Ecosystems, Inc. Apopka, FL, USA) three-shelf bench top system maintained at 28–30 °C. Daily husbandry procedures and housing conditions were as previously described^30,31^. Briefly, zebrafish were maintained on a 12 h–12 h light-dark cycle and fed dry fish pellets (New Life Spectrum Small Fish Formula, New Life International Inc., Homestead, FL, USA) to satiation once daily. On experimental trial days fish were fed after behavioural testing. The luminance in all of the testing arenas was (~32 cd/m^3^; cal SPOT photometer; Cooke Corp. CA, USA). All fish were naïve to the behavioural tests, and each fish was only used in one test.

### Drug Administration

Ethanol (Luxco, St. Louis, MO) was administered at 0.5, 1.0 or 1.5% in reverse osmosis (RO) water for 30 minutes. Scopolamine hydrobromide (Sigma-Aldrich, Oakville, ON) was dissolved in RO water to obtain concentrations of 200, 800, 1200, and 1600 µM. Scopolamine was administered to zebrafish by placing them in a 3-L dosage tank for 30 minutes. The dosage tank was filled with 1000 ml of drug solution, maintained at a temperature of 26–28 °C, was located adjacent the testing arena in the testing room and was surrounded by white corrugated plastic to prevent unwanted visual stimulation. Control fish for both ethanol and scopolamine comparisons underwent identical procedures without the presence of the drug. Fish were tested only once in control, ethanol, or scopolamine solutions. All solutions were made fresh each day of experimentation.

### Procedure

#### Novel approach test

Testing took place in a non-toxic plastic arena with a diameter of 34 cm, a circumference of 108.5 cm, and a depth of 15 cm as previously described^18^. The arena was filled to 6 cm with fresh habitat water every 5^th^ trial and maintained at a temperature of 26–28 °C. A 2 cm × 4.25 cm LEGO® figurine fixated in the center of the arena served as the novel object and was multicolored to avoid potential effects of innate color preferences^20,32^. Diffused lighting was used to ensure that behaviour was not affected by shadows cast in the arena. Zebrafish were individually netted from the dosing tank and released into the arena facing the object half way between the wall of the arena and the object. Behaviour was immediately recorded for 5 minutes using Ethovision XT (version 7.0, Noldus, VA, USA) with a Rainbow CCTV high resolution black and white CCD camera that was suspended 1 m above the experimental tank. The location and movement of the fish was recorded and quantified in Ethovision XT motion tracking software (version 7.0, Noldus, VA, USA). After the 5 minutes of video tracking, fish were netted out of the arena and placed in a separate holding tank before being returned to their habitat. The arena was divided into an inner and outer zone in Ethovision with a centered virtual circle with a diameter of 17 cm. The duration zebrafish spent in each zone was recorded in seconds, and locomotor activity was measured in terms of the distance traveled (cm) and immobility (s).

#### Novel tank diving test

Testing took place in a non-toxic glass arena with a height of 18 cm, a length of 25 cm, and a width of 5 cm. The tank was filled to 15 cm of habitat water and water was changed every 5^th^ trial. For each trial, individual fish were netted from a holding or dosing tank and were placed in the tank diving arena. Behaviour was immediately recorded for 10 minutes with Ethovision XT (version 9.0, Noldus, VA, USA). Previous research has shown that zebrafish engage in bottom-dwelling behaviour when first placed in the novel tank diving test and only begin to explore the upper zones after a few minutes of acclimation^21^. Because of this, the novel tank diving test can be used as an effective measure of zebrafish anxiety-like behaviour. Zebrafish also spend more time in the upper zone after chronic and acute ethanol administration^21,33,34^. For the analysis, the tank was divided equally into top, middle and bottom zones in Ethovision XT, and time spent in each zone was analyzed.

### Shoaling

Testing took place in a white, circular, non-toxic plastic arena with a diameter of 70 cm and was filled to 8 cm of habitat water. Water was changed every 3^rd^ trial. For each trial, six zebrafish were netted from their housing habitat and placed in a container with habitat water and a 400 micron spawning insert (Aquatic Habitats) for a 30 minute period before being placed into the experimental arena. The spawning insert holding the fish was lifted out of the container and carefully submerged in a dosing tank containing scopolamine or control solution. This procedure allowed for rapid and simultaneous transfer of all fish in a group^35^. Previous research has shown that anxious fish will swim closer together in a group compared to non-anxious fish^22,23^. Furthermore, zebrafish exposed to ethanol reliably swim farther apart, consistent with the anxiolytic effect of ethanol^22,36,37^. Behaviour was immediately recorded for 10 minutes with Ethovision XT (version 9.0, Noldus, VA, USA). The nearest neighbor distance (cm), and the inter-individual difference (cm) were calculated for each shoal^38^.

### Statistical analysis

Normality was tested using the D’Agostino-Pearson omnibus normality test or the Kolmogorov-Smirnov test with Dallal-Wilkinson-Lillefor P value test (for shoaling data with a small sample size). Unpaired two-tailed t-tests for parametric data and Mann-Whitney U tests and Kruskal-Wallis tests (with Dunn’s multiple comparison post hoc test) were used for non-parametric data. To calculate the EC_50_ value for the scopolamine dose-response in the novel approach test, data from 0 and up to 800 microM scopolamine was first normalized to a 0–100% scale, then log transformed and analyzed using a non-linear least square curve fit. All data was analyzed using Graphpad Prism 6.0 (CA, USA).

### Ethics Statement

All experiments were approved by the Grant MacEwan University Animal Research Ethics Board (AREB) under protocol number 05–12–13, in compliance with the Canadian Council for Animal Care (CCAC) guidelines for the care and use of experimental animals. The authors confirm that all experiments were were performed in accordance with these guidelines and regulations.

### Data Availability Statement

All data generated or analysed during this study are included in this published article (and its Supplementary Information files).

## Electronic supplementary material

Supplementary Information
