# Supplementary material for: Establishing zebrafish as a model to study the anxiolytic effects of scopolamine
Source: Sci Rep. 2017 Nov 8;7:15081. doi: 10.1038/s41598-017-15374-w (PMC5678162; doi:10.1038/s41598-017-15374-w)
Supplement: Supplementary file 1 — Supplementary Information [file 41598_2017_15374_MOESM1_ESM.pdf]

Supplementary Information
Raw Data

Title: Establishing zebrafish as a model to study the anxiolytic effects of scopolamine

Authors: Trevor J. Hamilton[1,2], Adam Morrill[1], Kayla Lucas[1], Joshua Gallup[1], Megan Harris[1], Meghan Healey[1], Taylor Pitman[1], Melike Schalomon[1], Shannon Digweed[1], Martin Tresguerres[3]

Affiliations:

[1]Department of Psychology, MacEwan University, Edmonton, AB, Canada, T5J 4S2.

[2]Neuroscience and Mental Health Institute, University of Alberta, Edmonton, AB, Canada, T6G 2H7.

[3]Scripps Institution of Oceanography, University of California San Diego, 9500 Gilman Drive, La Jolla, CA 92093

- Corresponding author: trevorjameshamilton@gmail.com

Ethanol Novel Approach Test

Time in inner zone (s)

control	Ethanol (%) 0.5	Ethanol (%) 1	Ethanol (%) 1.5
17.58425	2.23557	15.18185	5.305306
3.670335	33.19987	73.34	129.296
3.43677	8.875538	3.703705	58.92559
2.73607	24.19086	4.004004	17.28395
15.41541	4.104104	38.53854	28.86219
21.32132	2.335668	2.102102	48.71539
12.31231	9.009006	6.940272	14.38105
3.63697	0.734068	26.82682	0.834168
6.706706	2.569235	1.668335	50.28361
1.601601	22.98965	5.238574	103.637
11.44478	16.64998	10.91091	114.648
20.02002	2.102102	32.63264	1.568233
11.04438	3.036369	33.73373	36.3697
2.102102	3.370039	6.840173	77.44411
11.87854	2.936269	28.06139	19.15248
2.102103	9.609611		88.42175
1.334667			10.34368
4.070736			32.03203

Distance traveled (cm)

Control	0.5% Eth	1% Eth	1.5% Eth
1803.768	1690.91	5916.987	4676.352
4007.594	3544.085	3799.057	669.5609
3918.751	4659.136	959.7931	2422.881
3609.898	3245.185	5018.603	895.087
4349.391	2833.957	3194.693	4446.634
4462.484	3173.349	3596.961	3164.14
3437.224	3524.844	5772.05	214.1318
1895.332	3673.7	3849.808	880.8222
4936.032	2799.613	4784.581	4109.272

3958.141	2525.234	3617.839	2127.263
3934.875	2438.702	289.0891	2344.013
5803.09	3503.31	267.3674	3949.77
3305.879	4180.771	266.2663	2345.465
2903.709	2985.26	293.1598	3353.148
3376.344	3511.205	271.9386	1015.259
2842.222	3411.563		3118.37
3047.95			3436.525
4519.449			4470.25

Immobility (s)

Control	0.5% Eth	1% Eth	1.5% Eth
31.13113	49.24925	0	0.033366
0.233566	0.066734	1.101096	221.8552
0.100099	0.734068	226.3263	1.634962
0.100101	0.033367	0.066732	237.1705
0	5.305303	1.835165	10.14348
0	0.467133	104.2376	87.25392
78.61195	0.2002	0	271.2379
98.93228	0	0	206.1061
0	0.33367	0.467132	0.200197
75.74241	0.367034	1.468134	11.444779
0.734067	1.468137	0.133467	82.916253
0.4004	0.066734	0.033367	10.243571
0.433767	0.033367	0.100101	54.587905
0.467134	0.033366	4.170834	0.533867
0	2.736066	0.633966	147.180512
0.333667	0.133468		1.701703
1.868537			31.097768
0.033367			0.967637

Scopolamine - Novel Approach Test

Distance traveled (cm)

0	200	400	800	1200	1600
1803.768	4749.449	4425.048	2753.066	2961.461	3987.179
4007.594	3742.047	3413.206	5281.54	2340.645	3643.979
3918.751	3819.967	3191.863	3041.162	3273.709	3833.039
3609.898	3991.967	4187.806	3306.344	2896.832	2564.446
4349.391	3939.288	4290.92	4001.124	2406.522	3698.656
4462.484	2544.349	4794.614	3074.561	2119.555	6814.589
3437.224	4050.212	5831.062	2971.817	2059.05	4493.856
1895.332	3834.659	4095.795	2886.302	2062.471	4885.78
4936.032	3697.585	4406.611	1425.287	2698.972	4312.271
3958.141	3260.004	2024.506	3294.144	3147.392	2543.098
3934.875	4344.942	2834.971	4795.517	2609.544	3625.146
5803.09	4387.348	1884.504	5130.94	2194.28	3883.131
3305.879	3416.866	5403.369	2700.195	3465.891	4044.28
2903.709	5467.641	2885.529	4070.382	2992.198	2072.848
3376.344	2775.591	3688.948	3408.499	3572.342	2517.943
2842.222	5093.926	2454.214	7222.749	4873.429	4811.372
3047.95	4266.673		2145.414		2780.385
4519.449	6686.917		5275.024		4272.039
			7817.786		

Time in inner zone (s)

0	200	400	800	1200	1600
17.58425	7.27394	28.92893	9.209209	27.7611	20.65399
3.670335	0.633967	15.31532	11.77845	10.74408	3.003003
3.43677	11.67834	20.48716	27.29396	14.61462	13.41341

2.73607	7.874542	3.336669	23.69036	38.57191	31.36469
15.41541	19.65299	8.942276	13.27995	10.34367	14.18085
21.32132	12.37905	6.473141	68.7354	14.14748	11.21121
12.31231	54.88822	1.534868	17.51752	1.501502	28.02803
3.63697	1.301302	20.05338	37.50417	6.139473	9.943275
6.706706	7.073739	8.742076	27.49416	2.435769	9.676341
1.601601	2.702703	46.54655	17.88455	15.61562	5.905909
11.44478	9.309306	19.98665	10.51051	83.81715	8.57524
20.02002	26.99366	34.3677	23.89055	15.18185	14.38105
11.04438	13.88054	11.87854	13.61361	11.94528	14.71471
2.102102	18.28495	19.45278	10.51052	24.82483	30.4638
11.87854	3.470136	6.639973	12.67935	7.140474	25.45879
2.102103	1.768435	5.472139	25.39206	4.23757	0
1.334667	2.802805		15.04838		11.47815
4.070736	39.53955		5.13847		2.369035
			16.51652		

Immobility (s)

0	200	400	800	1200	1600
31.13113	0	0.600601	3.370041	0.6006	0
0.233566	0.133466	0.200199	0	0.367031	0
0.100099	0.166833	0.1001	0.433766	0.033366	2.2022
0.100101	0.600602	0	3.570233	0.166831	61.62828
0	0.166833	0.4004	0.233568	0.400401	0.200201
0	27.32733	0	22.95628	16.38305	0.100098
78.61195	16.51652	0	0.266931	7.507514	0.133467
98.93227	0	0.166835	0.433768	1.968638	0.200201
0	0.433766	0.3003	0.066734	0.033366	107.2406
75.74242	36.13614	3.536876	30.79747	34.20087	0.1001
0.734067	0.033367	2.1021	0	0.734068	0.133467
0.4004	0.033367	1.101105	17.68435	0.233568	0.300301
0.433767	0.6006	0.200198	60.89423	0.166835	6.539869
0.467134	0	0.800803	0.066734	0.200199	0.800801
0	0.400398	0.066734	0.033367	0.233568	0
0.333667	0	12.87954	0	1.134468	1.73507
1.868537	38.77211		4.104098		0.166834
0.033367	0.033366		0		0
			0		

Scopolamine Novel Tank Diving Test

Time in zone (s)

Top Zone

CTL	s
33.9673	345.7457
71.57158	241.5082
10.07674	177.3774
34.73473	104.004
75.1418	46.47982
107.2739	113.7137
41.44144	97.53087
142.4091	167.1672
1.001001	109.3427
40.20687	125.1251
	29.82983
	278.2783
	47.81449

Middle Zone

CTL	s
258.8922	190.7574
107.374	225.4254
72.03871	177.4441
203.6036	266.2663
149.5829	83.24991
94.4945	246.046
185.6857	158.8589
193.1598	189.623
74.74141	286.8869
184.5512	168.1682

	95.06172
	214.8148
	178.0447

Bottom Zone

CTL	s
307.1405	68.56857
421.0544	133.0998
517.8845	245.2119
361.6617	229.7631
375.2753	470.3036
398.2316	240.2736
372.8729	342.3757
264.4311	243.2432
524.2576	203.8038
375.2419	306.7401
	475.1418
	106.9403
	374.1742

Scopolamine
Shoaling

Interindividual distance (cm)

Control	Scopolamine
13.64534	21.58942
13.69688	21.36891
13.66039	20.75371
13.93193	20.54519
14.1509	21.39968
14.27142	20.85162

Nearest neighbour distance (cm)

Control	Scopolamine
10.91572	14.50113
8.516113	29.78
13.9546	16.9646
13.58025	18.48138
7.981942	18.96373
22.83022	19.00323
